# Going it Alone: A Scoping Review of Unbefriended Older Adults

**DOI:** 10.1017/S0714980817000563

**Published:** 2018-03

**Authors:** Stephanie Chamberlain, Sol Baik, Carole Estabrooks

**Affiliations:** 1 Faculty of Nursing, University of Alberta; 2 Department of Social Welfare, Seoul National University

**Keywords:** aging, scoping review, unbefriended, public guardianship, older adults, long term care, vieillissement, examen de portée, solitaire, tutelle publique, adultes âgés, soins de longue durée

## Abstract

Older adults who have reduced decision-making capacity and no family or friends to compensate for these deficiencies are known as *unbefriended* and require a public guardian. The purpose of this study was to review the peer-reviewed and grey literature to determine the scope of available research on unbefriended older adults in Canada and the United States. We found limited research examining unbefriended older adults. No Canadian studies or reports were located. Unbefriended older adults were childless or had fewer children, were more cognitively impaired, and were older than older adults who were not unbefriended. These findings demonstrate a stark scarcity of studies on unbefriended older adults. Research is urgently needed using standardized data collection of guardianship status in order to enable studies of the prevalence of public guardianship in Canada.

## RÉSUMÉ

Les personnes âgées dont la capacité de prise de décision est réduite et qui n’ont pas de soutien familial ou d’amis pour les aider à compenser ces déficits sont dites « solitaires » et nécessitent un tuteur public. Le but de cette étude était de recenser les publications avec comités de pairs et la littérature grise afin d’examiner la portée des travaux de recherches réalisés jusqu’à ce jour concernant les personnes âgées solitaires au Canada et aux États-Unis. Nous n’avons recensé que peu d’études sur ce sujet. Aucune étude ou rapport canadien n’a été trouvé. Les personnes âgées solitaires étaient plus avancées en âge, sans enfants ou avec peu d’enfants, et leurs troubles cognitifs étaient plus sévères, comparativement aux personnes âgées non solitaires. Ces résultats démontrent une rareté des études sur les personnes âgées solitaires. Il est urgent que davantage de recherches soient réalisées avec des collectes de données standardisées concernant la tutelle chez les personnes âgées solitaires afin de faciliter la réalisation d’études sur la prévalence de la tutelle publique au Canada.

Worldwide, the population is aging, with nearly 900 million people over the age of 60 (Alzheimer’s Disease International, 2015). As the population of older adults swells, so too does the prevalence of age-related diseases such as Alzheimer’s disease and other dementias (Alzheimer Society of Canada, 2010). Advanced age and cognitive impairment result in reduced decision-making ability (Boyle et al., 2012; Griffith, Dymek, Atchison, Harrell, & Marson, 2005; Kim, Karlawish, & Caine, 2002). Family members or friends may intervene and act as a guardian if an older adult is deemed incapable of managing his or her personal well-being and/or finances (Weisensee, Anderson, & Kjervik, 1996). However, not all older adults have a family member or friend available to act as their guardian. Changes in geographic mobility, family structure, childlessness (Albertini & Mencarini, 2014; Banks, Haynes, & Hill, 2009; de Medeiros et al., 2013), and being single for whatever reason (Barrett & Lynch, 1999; Wachterman & Sommers, 2006) have a negative impact on the availability of family members to act as guardians for older adults. Older adults are “unbefriended” if they lack decision-making capacity, lack an advanced directive and the ability to execute the directive, and lack a family member or a friend to act as their representative (Farrell et al., 2017; Karp & Wood, 2003; Pope & Sellers, 2012). The term *unbefriended* originated in the medical ethics literature and continues to be used as a term to denote any adult who does not have decision-making capacity, has no family or friends, or has family members or friends who are either unable or unwilling to assist with health decision-making (Bandy, Helft, Bandy, & Torke, 2010; Reynolds & Wilber, 1997). Unbefriended older adults require a public guardian. This article synthesizes the literature regarding unbefriended older adults, that is, those under public guardianship. Specifically, our scoping review describes the scope, study methods, geographic location of available empirical literature, and identifies characteristics (demographic, health) of unbefriended older adults.

## Guardianship

Principles of guardianship come from the legal tradition of *parens patriae,* the duty to protect persons who cannot care for themselves (Gillick, 1995; Iris, 1988; Schmidt, Bell, & Miller, 1981; Teaster, Schmidt, Abramson, & Almeida, 1999). Guardianship is a broad description of legal mechanisms that grant authority for managing personal and/or financial responsibility in the event an individual is incapacitated. Guardianship is one of the most restrictive actions that can be taken to limit legal rights (Lisi & Barinaga-Burch, 1995). It removes an individual’s right to vote, travel, determine own residence, or consent to medical treatment (Reynolds & Carson, 1999). Guardianship effectively *de-persons* the individual – removing them of all adult rights and responsibilities (Hightower, Heckert, & Schmidt, 1990; Schmidt, 1984; Teaster et al., 1999). Adults (18+) who are under guardianship are typically older, female, have multiple chronic conditions, and are socially isolated (Bandy et al., 2014; Doron, 2004; Reynolds, 1997a, 2002; Wilber, Reiser, & Harter, 2001).

Guardianship research does not always distinguish between different types of guardians. Guardians can be either private (family member or friend) or public (government, voluntary agency, paid service) (Teaster, Wood, Schmidt, & Lawrence, 2007). Public guardianship is the legal appointment of a public official or organization to assume decision-making responsibility when a family member or friend is either unavailable or unwilling (Teaster et al., 1999). Approximately 25–30 per cent of guardianship petitions are for public guardians, and the remainder are for a family member or friend (Bayles & McCartney, 1987; Bulcroft, Kielkopf, & Tripp, 1991; Lisi & Barinaga-Burch, 1995; Peters, Schmidt, & Miller, 1985; Teaster et al., 2005). Within the guardianship literature, public guardianship has received significantly less attention (Teaster et al., 2005). As a result, much less is known about individuals under public guardianship, which is troubling given that the restriction in autonomy as a result of public guardianship places individuals at heightened risk of abuse or neglect (Karp, 2006).

## Public Guardianship: United States and Canada

The role of public guardian varies based on the country of origin. In the United States, public guardians might be volunteers, agencies, or attorneys. In Canada, each province has their own Office of Public Guardians and/or Trustees and is typically associated with branches of provincial government. In England, the Office of the Public Guardian is an executive agency of the Ministry of Justice and will appoint panel deputies – typically lawyers and social service agencies – who act on behalf of the person who lacks capacity (Hartley-Jones, 2011). Although the concept of a public guardian exists in many countries, the research on public guardianship has been concentrated in the United States. Schmidt, Miller, Bell, and New (1981) conducted the first U.S. national study of public guardianship. They found the majority of persons with a public guardian were over age 65, female, low-income, and living in a long-term care facility or mental hospital (Schmidt, Miller, et al., 1981). Interest in unbefriended older adults emerged in the United States in the late 1980s following an investigative reporting series by the Associated Press (AP). At that time (1987), AP estimated that there were approximately 400,000 unbefriended older adults in the United States. Their reporting raised substantial concerns about the quality of care provided to unbefriended older adults, highlighting rampant ageism, abuse, and neglect (Bayles & McCartney, 1987). The AP series triggered nearly 20 years of reform and scholarship into the U.S. guardianship system. Currently, U.S. public guardianship programs are funded through some combination of court, state office, social service agency, or local municipality/county funding (Teaster et al., 2005). In the majority of U.S. states (*n* = 34), public guardianship programs are managed through a social service agency. Public guardianship through a social service agency introduces significant potential for conflict of interest. When an agency or program is both providing services and acting as a guardian and advocate, this could lead to unnecessary or undesired use of services by the person under guardianship (Teaster et al., 2005). On the other hand, it could result in the denial of necessary services when cost cutting is mandated.

Canada has a significantly different public guardianship system than the United States. Since Canada’s guardian and trustee system is managed at the provincial government level, it is akin to the U.S. independent state agency model. In Canada, three provincial Offices of the Public Guardian operate as special operating agencies or sole custodians (Manitoba, British Columbia, New Brunswick) under agreements with provincial departments. Operating as a special operating agency or sole custodian means that Offices function separately from the government, and these Offices can sue or be sued on behalf of clients; this organizational structure is meant to facilitate external monitoring and oversight.

The purpose of this scoping review was to review the peer-reviewed and grey literature to assess the scope of the available literature on unbefriended older adults. We aimed to describe the characteristics (demographic and health) of unbefriended older adults. In this article, we determine if Canadian literature exists and discuss implications for policy and practice.

## Methods

We conducted a scoping review to assess the types of evidence available and address the gaps in existing literature regarding unbefriended older adults (Arksey & O’Malley, 2005; Colquhoun et al., 2014). A scoping review was appropriate to address the range of available research on the topic of unbefriended older adults and enabled us to address the need for future research in this field of inquiry (Levac, Colquhoun, & O’Brien, 2010). A scoping review is a synthesis method used when the research question is broad in scope and contains a range of different study designs (Arksey & O’Malley, 2005; Armstrong, Hall, Doyle, & Waters, 2011; Rumrill, Fitzgerald, & Merchant, 2010). We conducted our scoping review based on the process developed by Arksey and O’Malley (2005) and later refined by Levac, Colquhon, and O’Brien (2010). The five stages of a scoping review as described by Arkey and O’Malley (2005) are as follows: (1) research question development, (2) literature search, (3) study selection, (4) data charting, and (5) data synthesis and summary. Our research question focused on a descriptive analysis of unbefriended older adults. We were unable to conduct quality assessments as was suggested by Levac et al. (2010) of the final included results due to highly disparate study designs and the descriptive nature of the final articles. We did include a section in our discussion describing necessary empirical directions for future research efforts and the utility of the research in policy and practice (Levac et al., 2010).

### Search Strategy

The search strategy and keywords were developed in consultation with a university health sciences librarian. The research librarian assisted in developing and refining the search strategy. We conducted the search using combinations and synonyms of the core concept keywords for “unbefriended” and “older adult”. We used the Boolean term “OR” when searching within core concepts, and “AND” to combine core concepts. An exemplary search strategy from the Medline database can be found in Table 1.

**Table 1:** Exemplary search strategy for Medline database (1946 to present) via OVID: Includes MEDLINE in-process & other non-indexed citations

#	Search Terms
1	exp Aged/
2	((gerontolog* or older adult* or elder* or senior* or geriatric* or aged). af.
3	1 and 2
4	exp Legal Guardians/
5	exp Decision Making/
6	exp Third-Party Consent/
7	(advocat$ or legal$ guardian$ or surrogate$ decision maker$ or decision making$ or no surrogate$ or incapacitate$ or unrepresent$ or public guardian$ or conservator$ or unbefriend$).mp
8	4 or 5 or 6 or 7
9	3 and 8
10	exp Health Behaviour/ or exp Health Status Indicators/ or exp Health Status/ or exp Health Services/ or exp Health Services for the Aged/
11	9 and 10
12	11 and 1991:2016. (sa_year)

### Study Inclusion and Exclusion Criteria

We included studies that were focused on unbefriended older adults. The study needed to include older adults who did not have a family or friend representative. We included only those studies that were available in English and published after 1991. We excluded studies that did not include older adults (defined here as those aged 60 and older). We excluded studies with mixed samples where data regarding the older adults could not be isolated from the larger sample. We excluded editorials, commentaries, and opinion articles.

The review was conducted from October to November 2016. We searched 12 electronic databases: Medline, CINAHL, PsycInfo, Cochrane Library, Abstracts in Social Gerontology, Family Studies Abstracts, Scopus, Web of Science Core Collection, PubMed, Social Work Abstracts, SocINDEX, and Legal Source. Grey literature sources included ProQuest dissertations and relevant conference programs (e.g., Gerontological Society of America Annual Conference, Canadian Association on Gerontology Annual Conference, National Conference on Guardianship, World Congress on Adult Guardianship). We searched the grey literature with the same search terms. All search results were exported and stored in Zotero, an online citation software program. Once the searches from each database were completed and compiled, all duplicates were removed. We completed ancestry searches of all the full-text paper reference lists.

### Study Selection

We considered studies that described the characteristics (e.g., age, socioeconomic status, social support) or health of unbefriended older adults. We defined a person who was unbefriended (also described as a ward, or conservatee in the literature), as someone unable to meet their own personal health needs and/or manage the essential aspects of personal financial resources, and who had no willing or able family member or friend to act as their guardian (Hightower et al., 1990). We conducted a two-stage study selection process. In the first stage, two authors of our study (SC and SB) reviewed article titles and abstracts to assess if the article met the identified inclusion and/or exclusion criteria, or if the full-text study was needed to determine study applicability. Both reviewers labelled an abstract to include for further review as either “Yes”, “No”, or “Unsure”. Discrepancies were resolved by consensus, and all titles without an abstract or abstracts labelled as Unsure were carried forward to the full-text review. The second stage consisted of two team members (SC and SB) independently reviewing all of the full-text articles.

To begin, the reference lists from the full-text articles and grey literature were searched for articles not yet included in the review. Differences in the decision to include a study for full-text review were resolved by team discussion and consensus. Further review of the full-text articles in relation to the inclusion and exclusion criteria led additional articles to be rejected before data charting. Two team members separately charted the data from the final included studies and then came together to determine the appropriate information to be extracted from the studies. Authors SC and SB completed the data extraction and synthesis. We analysed a final number of five articles. A summary of the collected information from the full text articles – including authorship, study design, setting/location, sample/subjects, number under guardianship in study sample, older adult characteristics, comparison group (if any), and statistical analysis – can be found in Table 2.

**Table 2:** Study characteristics

Author, Journal, Year	Study Design	Setting, Location	Sample, Subjects	Proportion of OA without Family or Friend Guardian in Total Sample	Sample Demographic Characteristics	Statistical Analysis
Janofsky, *Journal of Geriatric Psychiatry & Neurology*, 1993	Cross-sectional survey	Nursing home (*n* = 1) Country: USA	Emergency contact or family member of resident (*n* = 191 respondents)	*n* = 63 residents lacked durable power of attorney or guardian Prevalence = 33%	*n* = 46 (73%) were not considered mentally capable of decision-making *n* = 16 (25.4%) were considered mentally capable of decision-making	None
Teaster, dissertation, 1997	Qualitative interviews, document review	Qualitative interviews, document review of court petitions, and case files from older adults under public guardianship Country: USA	Case files from older adults included in qualitative interviews and observations (*n* = 19 )	NA	*n* = 10 (52.6%) able to communicate verbally *n* = 9 (47.4%) unable to communicate verbally Average age = 80.7 years *n* = 13 (68.4%) lived in a nursing home, 1 (5.5%) in hospital, 1 (5.3%) in own home, 3 (15.8%) in group home, 1 (5.5%) home for adults *n* = 10 (52.6%) have a dementia All had at least one major medical diagnosis; the average number of major medical diagnoses was 3+	NA
**Author, Journal, Year**	**Study Design**	**Setting, Location**	**Sample, Subjects**	**Proportion of OA without Family or Friend Guardian in Total Sample**	**Sample Demographic Characteristics with Comparison Groups**	**Statistical Analysis**
Reynolds, *Aging and Mental Health*, 1997^a^	Cross-sectional survey	Records from Los Angeles County Department of Mental Health’s Office of Public Guardian Country: USA	Wards who were 70+ (*n* = 623) Total # of records: *n* = 2,151	*n* = 623 older adults under public guardianship Prevalence = 29%	**Public Conservatees vs. Nationally Representative Sample of Older Adults** Age 85+: Public = 30.2%; National = 13.6% Married: Public = 6%; National = 54.9% Single: Public = 38%; National = 2.9% Separated or divorced: Public = 19.8%; National = 5% Childless: Public = 73.2%; National = 14.9% No Siblings: Public = 71.3%; National = 23.6% High School Graduate +: Public = 65.1%; National = 55.9% Wealth (None): Public = 31.9%; National = 6.5% Real estate value (None): Public = 83.2%; National = 25.5%	Pearson χ^2^ *p* < .05 reported
Reynolds, *Aging and Mental Health*, 1999	Cross-sectional survey	Court records in two counties Country: USA	*n* = 406 court files	*n* = 167 had family guardians *n* = 147 had professional guardians Prevalence = 41%	**Public Conservatees vs. Family Conservatees** Age (mean): Public = 74.26; Family = 63.45 With Spouse (Yes): Public = 7.60%; Family = 20.2% Number of children: Public = 0.29; Family = 0.96 Number of siblings: Public = 0.41; Family = 0.71	Pearson χ^2^ *p* < .05 reported
Reynolds, *Research on Aging*, 1997	Cross-sectional survey	Records from Los Angeles County Department of Mental Health’s Office of Public Guardian Country: USA	Wards who were 60+ (*n* = 894) Total # of records: *n* = 2,118	42.2%	**Older (60+) vs. Young (<60)** Female: Old = (0.63); Young = (0.33) Married: Old = (0.06); Young = (0.04) Single: Old = (0.39); Young = (0.74) Widowed: Old = (0.24); Young = (0.01) Divorced/Separated: Old = (0.29); Young = (0.18) Indications of Severe Disability: Old = (0.35); Young = (0.14) ADL Impairments: Old = (2.56); Young = (0.14) Diagnosis of schizophrenia or other psychosis: Old = (0.28); Young = (0.74) Diagnosis of dementia/OBS: Old = (0.46); Young = (0.05)	No tests for significant differences; Conducted regression model with age as predictor of placement in locked facility

OA = older adults

OBS = organic brain syndrome

a The comparative data set of a nationally representative sample of older adults came from the Asset and Health Dynamics Among the Oldest Old (AHEAD) study described here: https://www.aeaweb.org/rfe/showRes.php?rfe_id=72&cat_id=5

## Results

Our search yielded 14,793 articles once duplicates were removed. After title and abstract screening, we assessed 185 full-text articles. We excluded 180 articles because they did not meet the review criteria. Of all the studies that we excluded, the largest number (*n* = 62) were excluded because they focused on family and friend guardians and not public guardians. We excluded (*n* = 43) studies because they did not provide any description of demographic characteristics or health outcomes. Studies were excluded because they did not provide any empirical research; rather, they discussed the challenges and legal implications of public guardianship (*n* = 21). We excluded (*n* = 17) studies that described the characteristics and health outcomes of unbefriended adults; however, information about the older adult participants could not be isolated from the larger sample. We excluded three studies because the full text was not available in English (Japanese = 2, German = 1).

**Figure 1:**
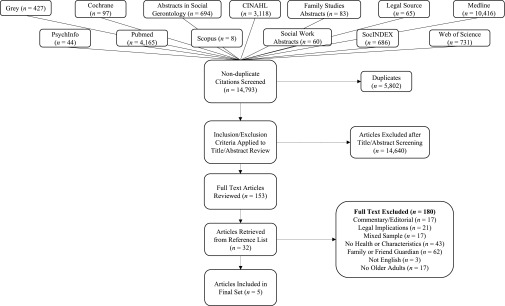
Search strategy included and excluded studies

Figure 1 shows the search, screening, and final selection process. Our search and review resulted in a final total of five papers that matched our review criteria.

Of the five articles included in the final sample, one study was conducted on long-term care, one reported on data collected from state or county legal records, and three collected information from a state or regional office of the public guardian. All of the included studies were conducted in the United States and were published between 1993 and 1999.

## Discussion

Our scoping review of unbefriended older adults revealed an exceptionally small body of peer-reviewed and grey literature. Three studies used information collected from county legal records and case files; however, they varied in state of origin. We found only one study that included older adults from long-term care, which provided minimal description of the characteristics of those residents without a family or friend guardian (Janofsky & Rovner, 1993). Our results indicated that between 29 and 42 per cent of older adults in the study samples were unbefriended.

### Characteristics of Unbefriended Older Adults

Our findings suggest a grim picture of unbefriended older adults. They are more likely to be single, childless, have fewer siblings, and limited financial resources when compared to older adults with a family or friend guardian (Reynolds & Carson, 1999; Reynolds & Wilber, 1997). Unbefriended older adults often have a diagnosis of a dementia or related cognitive impairment and multiple chronic diseases (Janofsky & Rovner, 1993; Reynolds, 1997b; Teaster, 1997).

Our scoping review results suggest that at this time there is little value added by (nor adequate) literature with which to conduct a systematic review on the characteristics or health of unbefriended older adults. Empirical research is extremely limited. Studies included in the review demonstrate disparate methods and outcome measures that leave us unable to make any meaningful comparisons between the studies. Our results emphasize the erratic and sparse literature base for this population of unbefriended individuals.

Our findings reveal an alarming lack of data on those residents who are unbefriended and living in institutional settings such as long-term care. Public guardianship imposes significant limitations on the older adult’s ability to decide location of residence, and when coupled with mental and physical limitations, means these individuals are likely to live in a long-term care facility (Reynolds, 1997b; Teaster, 1997). Although research reports indicate that once older adults are placed under public guardianship they are more likely to be transferred to long-term care (Menio, Halperin, Campbell, & Reever, 2013; Reynolds, 1997b; Reynolds & Carson, 1999), we found only one study specifically examining long-term care residents (Janofsky & Rovner, 1993).

Research efforts that examine the health and care provided to unbefriended older adults should be directed at LTC facilities (Teaster et al., 2007). However, since no state or provincial records indicate location of residence, we are unable to discern who is providing care to this vulnerable population and if there are gaps in quality of care. Unbefriended older adults are exceptionally vulnerable to poor quality of care due to inadequate family or friend support (Cohen, Wright, Cooney, & Fried, 2015; Effiong & Harman, 2014). Without reliable information on the location of residence for these older adults, we are unable to identify who is providing their care and if they are receiving quality care. Farrell et al. (2017) recommended that future research is needed to better quantify the number of unbefriended older adults across different care settings (e.g., community, acute, long-term care). Limited empirical research and an inability to track the location of residence for unbefriended older adults reflects a significant gap in our knowledge and an opportunity for future research that would inform policy.

Our review located *no Canadian studies or reports*. Since our review found no Canadian studies or reports on the characteristics or health of unbefriended older adults, we have no idea how Canada may or may not compare to the United States. Discussions with several provincial policy analysts from the Office of the Public Guardian in Alberta suggest that the Canadian population of unbefriended older adults likely does not differ substantially from those in the United States. However, given our lack of reporting on these older adults in Canada, we are unable to substantiate these claims or make meaningful comparisons. Recently, the Office of the Public Guardian in the Northwest Territories made national news for its extended waiting periods (up to a year) for guardianship applications and inadequate resources to deal with growing caseloads (Gleeson, 2016). Reports from Offices of the Public Guardian in New Brunswick and Nova Scotia indicate that, with their current budgets and staffing levels, they are unable to cope with any increase in demand for guardianship services (Doherty, 2015; Theriault, 2014). Further inquiry is imperative to establish the number of older adults requiring guardianship services in Canada and prepare the social service system for the growing aging population.

All of the included studies that described the characteristics or health of unbefriended older adults were cross-sectional. Future research should focus on longitudinal assessments of health and identifying unmet care needs of this population of older adults (Reynolds, 1997b). Our findings raise important questions not answered by the available literature. There is an obvious and troubling gap in the research regarding unbefriended older adults and their unmet care needs. Research in the 1980s and 1990s suggests that these individuals have limited contact with their public guardian (Schmidt, Miller, Peters, & Loewenstein, 1988). In the past 20 years, little insight has been gained on the frequency or quality of interactions between the public guardian and the individual under guardianship, or its influence on quality of life and quality of care. National reports in the United States indicated that guardians have enormous and variable caseloads (even as high as one guardian for 341 persons under guardianship (Schmidt, Bell, et al., 1981; Teaster et al., 2007). Linking information about caseload, visiting frequency, and type of guardianship activity with individual health outcomes is an essential step in determining appropriate policy and practice recommendations.

### Implications for Policy and Practice in Canada

In both the United States and Canada, a lack of state or provincial coordination has resulted in variability in (negligible) national reporting and inconsistent regional oversight. Results of this review demonstrate that those unbefriended older adults may have a number of health and social limitations, potentially leading to poor quality of life. Given the provincial administration of public guardianship and the challenges of collecting even basic demographic information, analysis of health and quality of care of unbefriended older adults could focus on those already available sources of data. Throughout most of Canada, the Resident Assessment Data Minimum Data Set (RAI-MDS 2.0) is used to routinely collect personal and health information about residents living in long-term care. This instrument offers an opportunity to assess the prevalence and health outcomes of residents who do not have family or friend guardians. The items that assess the presence of family or public trustee are not mandatory to complete, resulting in an underestimation of unbefriended older adults in the RAI-MDS. Although the RAI-MDS likely underestimates unbefriended LTC residents, it is collected across Canada and could allow us to examine unbefriended residents’ clinical and functional status, which is currently not possible with data collected by provincial Offices of the Public Guardian.

Offices of the Public Guardian can serve in a variety of substitute decision-making roles, not only as public guardians but also as powers of attorney, trustee, and other more limited decision-making capacity roles. Future research could examine the different types of guardianship and link it with demographic characteristics and services to determine if there are groups who are using certain services with greater or lesser intensity. This would contribute to improved organizational planning and policies that reflect the groups that are most frequently using various guardianship services.

### Strengths and Limitations

Our scoping review was completed with the assistance of a health science research librarian. We conducted an ancestry analysis from the full text, peer-reviewed articles and reports to ensure that all available literature was reviewed. We were limited to English-language publications, and as a result excluded three studies. Title assessment and abstract assessment was restricted to guardianship and older adults, which may have limited articles that were not explicit about their population. Guardianship models and terminology vary among different states and countries (Teaster et al., 2007). If a paper did not explicitly describe the guardianship status as public or a situation where an individual did not have a family member or friend guardian, we were unable to include it in our findings. We did not report demographic characteristics for samples that were not specifically described for older adults.

## Conclusion

We found limited peer-reviewed literature describing the prevalence and characteristics of unbefriended older adults. All of the literature concerning public guardianship was U.S. based. This review reveals troubling gaps in the reporting of guardianship status. This is a population that is likely to grow, and longitudinal studies on health and care needs are needed to examine the potential health impact of unbefriended older adults. Without studies of characteristics or health outcomes, we are unable to adapt our continuing care to meet the needs of this unique population. Although this group of older adults – the unbefriended – arguably constitutes the highest risk group of older adults, there is no population-level data on this population in Canada.

## References

[ref1] Albertini M., & Mencarini L. (2014). Childlessness and support networks in later life: New pressures on familistic welfare states? Journal of Family Issues, 35(3), 331–357.

[ref2] Alzheimer Society of Canada. (2010). *Rising tide: The impact of dementia on Canadian society* Toronto, ON. Retrieved from http://www.alzheimer.ca/∼/media/Files/national/Advocacy/ASC_Rising_Tide_Full_Report_e.pdf

[ref3] Alzheimer’s Disease International. (2015). *World Alzheimer report 2015: The global impact of dementia* London, ENG. Retrieved from https://www.alz.co.uk/research/WorldAlzheimerReport2015.pdf

[ref4] Arksey H., & O’Malley L. (2005). Scoping studies: Towards a methodological framework. International Journal of Social Research Methodology, 8(1), 19–32. doi:10.1080/1364557032000119616

[ref5] Armstrong R., Hall B. J., Doyle J., & Waters E. (2011). Cochrane Update. ‘Scoping the scope’ of a Cochrane review. Journal of Public Health, 33(1), 147–150. doi:10.1093/pubmed/fdr015 21345890 10.1093/pubmed/fdr015

[ref6] Bandy R., Helft P., Bandy R., & Torke A. (2010). Medical decision-making during the guardianship process for incapacitated, hospitalized adults: A descriptive cohort study. Journal of General Internal Medicine, 25(10), 1003–1009. 20422304 10.1007/s11606-010-1351-8 PMC2955482

[ref7] Bandy R., Sachs G., Montz K., Inger L., Bandy R., & Torke A. (2014). Wishard Volunteer Advocates Program: An intervention for at-risk, incapacitated, unbefriended adults. Journal of the American Geriatrics Society, 62(11), 2171–2179. doi:10.1111/jgs.13096 25354983 10.1111/jgs.13096

[ref8] Banks L., Haynes P., & Hill M. (2009). Living in single person households and the risk of isolation in later life. International Journal of Ageing and Later Life, 4(1), 55–86. doi:10.3384/ijal.1652-8670.094155

[ref9] Barrett A. E., & Lynch S. M. (1999). Caregiving networks of elderly persons: Variation by marital status. The Gerontologist, 39(6), 695–704. 10650679 10.1093/geront/39.6.695

[ref10] Bayles F., & McCartney S. (1987). *Guardians of the elderly: An ailing system Part 1: Declared ‘legally dead’ by a troubled [system]* Retrieved from http://www.apnewsarchive.com/1987/Guardians-of-the-Elderly-An-Ailing-System-Part-I-Declared-Legally-Dead-by-a-Troubled-System/id-1198f64bb05d9c1ec690035983c02f9f

[ref11] Boyle P. A., Yu L., Wilson R. S., Gamble K., Buchman A. S., & Bennett D. A. (2012). Poor decision making is a consequence of cognitive decline among older persons without Alzheimer’s disease or mild cognitive impairment. PLoS ONE, 7(8), e43647. doi:10.1371/journal.pone.0043647 10.1371/journal.pone.0043647 PMC3423371 22916287

[ref12] Bulcroft K., Kielkopf M. R., & Tripp K. (1991). Elderly wards and their legal guardians: Analysis of county probate records in Ohio and Washington. The Gerontologist, 31(2), 156–164. 2044987 10.1093/geront/31.2.156

[ref13] Cohen A. B., Wright M. S., Cooney L. Jr., & Fried T. (2015). Guardianship and end-of-life decision making. JAMA Internal Medicine, 175(10), 1687–1691. doi:10.1001/jamainternmed.2015.3956 26258634 10.1001/jamainternmed.2015.3956 PMC4683611

[ref14] Colquhoun H. L., Levac D., O’Brien K. K., Straus S., Tricco A. C., Perrier L., … Moher D. (2014). Scoping reviews: Time for clarity in definition, methods, and reporting. *J* Journal of Clinical Epidemiology, 67(12), 1291–1294. doi:10.1016/j.jclinepi.2014.03.013 10.1016/j.jclinepi.2014.03.013 25034198

[ref15] de Medeiros K., Rubinstein R. L., Onyike C. U., Johnston D. M., Baker A., McNabney M., … Samus Q. M. (2013). Childless elders in assisted living: Findings from the Maryland Assisted Living Study. Journal of Housing for the Elderly, 27(1/2), 206–220. 24729653 10.1080/02763893.2012.754823 PMC3977594

[ref16] Doherty D. (2015). *Annual report: New Brunswick Legal Aid Services Commission public trustee services* Fredericton, NB. Retrieved from http://www.legalaid.nb.ca/en/uploads/file/PT%20Annual%20Report%20and%20FS%202014-15%20Bilingual.pdf

[ref17] Doron I. (2004). Aging in the shadow of the law: The case of elder guardianship in Israel. Journal of Aging & Social Policy, 16(4), 59–77. Retrieved from https://www.ncbi.nlm.nih.gov/pubmed/15724573 15724573 10.1300/J031v16n04_04

[ref18] Effiong A., & Harman S. (2014). Patients who lack capacity and lack surrogates: Can they enroll in hospice? Journal of Pain and Symptom Management, 48(4), 745–750. e741. doi:10.1016/j.jpainsymman.2013.12.244 24709366 10.1016/j.jpainsymman.2013.12.244

[ref19] Farrell T. W., Widera E., Rosenberg L., Rubin C. D., Naik A. D., Braun U., … the Ethics, Clinical Practice and Models of Care Public Policy Committees of the American Geriatrics, Society. (2017). AGS position statement: Making medical treatment decisions for unbefriended older adults. Journal of the American Geriatrics Society, 65(1), 14–15 (e11-e15). doi:10.1111/jgs.14586 10.1111/jgs.14586 27874181

[ref20] Gillick M. R. (1995). Medical decision-making for the unbefriended nursing home resident. Journal of Ethics, Law, & Aging, 1(2), 87–92. 11654399

[ref21] Gleeson R. (2016). *Backlog at N.W.T. public guardian’s office ‘a problem,’ says health minister. CBC News* Retrieved from http://www.cbc.ca/news/canada/north/nwt-public-guardian-backlog-1.3586088

[ref22] Griffith H. R., Dymekval-Valentine M. P., Atchison P., Harrell L. E., & Marson D. C. (2005). Medical decision-making in neurodegenerative disease: Mild AD and PD with cognitive impairment. Neurology, 65(3), 483–485. doi:10.1212/01.wnl.0000171346.02965.80 16087924 10.1212/01.wnl.0000171346.02965.80

[ref23] Hartley-Jones P. (2011). The role of the Office of the Public Guardian in investigations of abuse. Journal of Adult Protection, 13(3), 160–166. doi:10.1108/14668201111160750

[ref24] Hightower D., Heckert A., & Schmidt W. (1990). Elderly nursing home residents’ need for public guardianship services in Tennessee. Journal of Elder Abuse & Neglect, 2(3–4), 105–122. doi:10.1300/J084v02n03_07

[ref25] Iris M. A. (1988). Guardianship and the elderly: A multi-perspective view of the decision-making process. The Gerontologist, 28, 39–45. 3169593 10.1093/geront/28.suppl.39

[ref26] Janofsky J. S., & Rovner B. W. (1993). Prevalence of advance directives and guardianship in nursing home patients. Journal of Geriatric Psychiatry & Neurology, 6(4), 214–216. 8251049 10.1177/089198879300600406

[ref27] Karp N. (2006). Federal options to improve America’s ailing guardianship system: A white paper for the Senate Special Committee on Aging. Washington, DC: AARP Retrieved from http://assets.aarp.org/rgcenter/consume/m_2_guardianship.pdf

[ref28] Karp N., & Wood E. (2003). Incapacitated and alone: Health care decision-making for the unbefriended elderly (Report: 1-59031-272-4). Washington, DC: American Bar Association Commission on Law and Aging Retrieved from http://www.americanbar.org/content/dam/aba/administrative/law_aging/2003_Unbefriended_Elderly_Health_Care_Descision-Making7-11-03.authcheckdam.pdf

[ref29] Kim S. Y. H., Karlawish J. H. T., & Caine E. D. (2002). Current state of research on decision-making competence of cognitively impaired elderly persons. The American Journal of Geriatric Psychiatry, 10(2), 151–165. 11925276

[ref30] Levac D., Colquhoun H., & O’Brien K. K. (2010). Scoping studies: Advancing the methodology. Implement Science, 5, 69. doi:10.1186/1748-5908-5-69 10.1186/1748-5908-5-69 PMC2954944 20854677

[ref31] Lisi L. B., & Barinaga-Burch S. (1995). National study of guardianship systems: Summary of findings and recommendations. Clearinghouse Review, 29(6), 643–653.

[ref32] Menio D., Halperin A., Campbell J., & Reever K. (2013). *The state of guardianship in Pennsylvania: Results from the 2012 CARIE study of guardianship in the Commonwealth of Pennsylvania* Retrieved from https://www.carie.org/wp-content/uploads/2013/11/CARIE-Guardianship-Study.pdf

[ref33] Peters R., Schmidt W. C., & Miller K. S. (1985). Guardianship of the elderly in Tallahassee, Florida. The Gerontologist, 25(5), 532–538. 4065656 10.1093/geront/25.5.532

[ref34] Pope T. M., & Sellers T. (2012). The unbefriended: Making healthcare decisions for patients without surrogates (Part 1). Journal of Clinical Ethics, 23(1), 84–96. 22462389

[ref35] Reynolds S. L. (1997a). Criteria for placing older adults in public conservatorship: Age as proxy for need. The Gerontologist, 37(4), 518–526. 9279041 10.1093/geront/37.4.518

[ref36] Reynolds S. L. (1997b). Protected or neglected: An examination of negative versus compassionate ageism in public conservatorship. Research on Aging, 19(1), 3–25. http://dx.doi.org/10.1177/0164027597191001

[ref37] Reynolds S. L. (2002). Guardianship primavera: A first look at factors associated with having a legal guardian using a nationally representative sample of community-dwelling adults. Aging & Mental Health, 6(2), 109–120. doi:10.1080/13607860220126718 12028879 10.1080/13607860220126718

[ref38] Reynolds S. L., & Carson L.D. (1999). Dependent on the kindness of strangers: Professional guardians for older adults who lack decisional capacity. Aging & Mental Health, 3(4), 301–310.

[ref39] Reynolds S. L., & Wilber K. H. (1997). Protecting persons with severe cognitive and mental disorders: an analysis of public conservatorship in Los Angeles County, California. Aging & Mental Health, 1(1), 87–97.

[ref40] Rumrill P. D., Fitzgerald S. M., & Merchant W. R. (2010). Using scoping literature reviews as a means of understanding and interpreting existing literature. Work, 35(3), 399–404. doi:10.3233/wor-2010-0998 20364059 10.3233/WOR-2010-0998

[ref41] Schmidt W. (1984). The evolution of a public guardianship program. Journal of Psychiatry & Law, 12(3), 349–372.

[ref42] Schmidt W., Bell W., & Miller K. (1981). Public guardianship and the elderly: Findings from a national study. The Gerontologist, 21(2), 194–202. 7215895 10.1093/geront/21.2.194

[ref43] Schmidt W., Miller K., Bell W., & New E. (1981). Public guardianship and the elderly. Cambridge, MA: Ballinger. 10.1093/geront/21.2.194 7215895

[ref44] Schmidt W., Miller K., Peters R., & Loewenstein D. (1988). A descriptive analysis of professional and volunteer programs for the delivery of public guardianship services. Probate Law Journal, 8(2), 125–156.

[ref45] Teaster P. B. (1997). *When the state takes over a life: The public guardian as public administrator* (Doctoral dissertation). Virginia Polytechnic Institute and State University, Blacksburg, VA. Retrieved from https://theses.lib.vt.edu/theses/available/etd-36171339701021/unrestricted/GUARDIAN.PDF

[ref46] Teaster P. B., Schmidt W. C., Abramson H., & Almeida R. (1999). Staff service and volunteer staff service models for public guardianship and “alternatives” services: Who is served and with what outcomes? Journal of Ethics, Law & Aging, 5(2), 131.

[ref47] Teaster P. B., Wood E. F., Lawrence S. A., & Schmidt W. C. (2005). Wards of the state: A national study of public guardianship. Stetson Law Review, 37 Retrieved from http://www.stetson.edu/law/lawreview/media/wards-of-the-state-a-national-study-of-public-guardianship.pdf

[ref48] Teaster P. B., Wood E. F., Schmidt W. C. Jr., & Lawrence S. A. (2007). Public guardianship after 25 years: In the best interest of incapacitated people? Washington, DC: American Bar Association.

[ref49] Theriault M. (2014). Public trustee annual report. Halifax, NS: Office of the Public Trustee.

[ref50] Wachterman M. W., & Sommers B. D. (2006). The impact of gender and marital status on end-of-life care: Evidence from the National Mortality Follow-Back Survey. Journal of Palliative Medicine, 9(2), 343–352. 16629564 10.1089/jpm.2006.9.343

[ref51] Weisensee M. G., Anderson J. B., & Kjervik D. K. (1996). Family members’ retrospective views of events surrounding the petition for a conservatorship or guardianship. Journal of Nursing Law, 3(3), 19–30.

[ref52] Wilber K., Reiser T., & Harter K. (2001). New perspectives on conservatorship: The views of older adult conservatees and their conservators. Aging, Neuropsychology & Cognition, 8(3), 225–240.

